# Global Properties and Functional Complexity of Human Gene Regulatory Variation

**DOI:** 10.1371/journal.pgen.1003501

**Published:** 2013-05-30

**Authors:** Daniel J. Gaffney

**Affiliations:** Wellcome Trust Sanger Institute, Cambridge, United Kingdom; University of Michigan, United States of America

## Abstract

Identification and functional interpretation of gene regulatory variants is a major focus of modern genomics. The application of genetic mapping to molecular and cellular traits has enabled the detection of regulatory variation on genome-wide scales and revealed an enormous diversity of regulatory architecture in humans and other species. In this review I summarise the insights gained and questions raised by a decade of genetic mapping of gene expression variation. I discuss recent extensions of this approach using alternative molecular phenotypes that have revealed some of the biological mechanisms that drive gene expression variation between individuals. Finally, I highlight outstanding problems and future directions for development.

## Introduction

Mammalian genomes harbour a diverse array of gene regulatory elements. Fuelled by rapid technological advances, a decade of genomics research has begun to reveal the location, diversity, and richness of the regulatory fraction of the human genome [1]. However, the impact of naturally occurring genetic regulatory variation on downstream cellular and organismal phenotypes is not well understood. As a result, human genomics is becoming increasingly focused on characterising interindividual regulatory variation. In parallel, genome-wide association studies (GWASs) have also highlighted the importance of regulatory polymorphisms in driving human phenotypic variation [2]. To realise the full potential of association studies, human disease geneticists are also turning their attention to the functional interpretation of regulatory variation.

Studies that combine genetic mapping with characterisation of molecular and cellular traits, sometimes referred to as genetical genomics [3] or cellular genomics [4], enable identification of regulatory variation on a genome-wide scale. This study design features simultaneous assaying of cellular traits in multiple individuals followed by mapping of genetic correlates using linkage or association analysis. Because many phenotypes and individuals are assayed simultaneously, genetical genomics is a powerful method for observing global properties of regulatory variation [5]. Additionally, molecular or cellular QTLs can connect complex phenotypes such as disease susceptibility with low-level molecular changes like gene expression or transcription factor binding [6], [7].

Cellular mRNA levels are one of the most readily accessible cellular phenotypes, both by microarray hybridisation and, more recently, via high throughput sequencing. The first genome-wide study of natural genetic variation in gene expression was attempted in budding yeast [8] and was quickly followed by work in mice [9] and humans [10]–[12]. These seminal early studies highlighted the power of genetical genomics for detecting and interpreting gene regulatory variation, and introduced lymphoblastoid cell lines (LCLs) as a model system for understanding this variation in humans. Since then, eQTL maps have been generated in a broad range of taxa, cell lines, primary tissues, and population cohorts. In this review, I will discuss the global properties of regulatory variation that have emerged from a decade of eQTL mapping studies. I will focus on recent extensions of genetic mapping to a rich variety of alternative molecular phenotypes and the functional insights these have provided. Finally, I will summarise future challenges and areas for development.

## Biological Properties of Regulatory Variation: Insights from eQTL Studies

### Heritability and Genetic Architecture of Gene Expression

In humans, expression levels of between 40% and 90% of genes are significantly heritable across a range of tissues [13]–[15] with median heritability estimates ranging from approximately 15%–35% [11], [13]–[18]. The genetic architecture of gene expression is relatively complex, with an average eQTL explaining a small to moderate fraction of additive genetic variance, from 27% in yeast [19], <40% in rats [20], and between 5% and 18% in humans for SNPs [15], [16], [21] and 9% and 18% for CNVs [22]. Thus, genetic control of transcription at most genes appears to be polygenic. These effect sizes are, however, substantially larger than for typical whole-organism traits, and association signals in eQTL mapping studies often exceed standard genome-wide thresholds of significance, despite sample sizes as small as 50 individuals [23]. Regulatory variation also appears widespread in humans, with eQTLs frequently detected at hundreds or thousands of loci [23]. Given the small sample sizes of most eQTL studies, it seems highly likely that large numbers of small-effect eQTLs remain to be discovered. The genetics of gene expression also appears to be complex, with evidence for pleiotropy, epistasis, genotype environment interaction, and transgressive segregation (for a review, see [23]). Population context may also play some role in determining eQTL magnitude and direction, although this seems to be a relatively rare occurrence [24].

A genetic variant may influence the expression of one or both alleles of a transcript, referred to as regulation *cis* or *trans*, respectively. *cis*-acting variation is frequently assumed to be located close to the regulated gene, while *trans*-acting polymorphisms operate distally, perhaps on another chromosome. For the most part, this assumption is supported by studies of allele-specific expression [25], [26]. The relative importance of *cis* and *trans* modes of regulation has been widely debated in the literature. To date, the majority of eQTLs detected have been located relatively close to the regulated gene and probably function in *cis*, while convincing *trans-*eQTLs have been much more difficult to detect (see below). Indeed, many studies have ignored *trans-*eQTLs altogether, and restricted their search window to regions proximal to the expressed gene. *cis-*eQTLs show a characteristic distribution around genes and are strongly enriched close to transcription start sites (TSSs) and within gene bodies [24], [27], [28]. However, eQTLs near the TSS are not easily explained at the sequence level and do not appear to be strongly enriched in some canonical core promoter motifs such as the TATA box, although they show weak enrichment in other element types [29]. Perhaps surprisingly, eQTLs are overrepresented within exons [27]. In microarray data, at least some of this signal emanates from alternative splicing of the exon to which an array probe hybridises, usually located in the 3′ UTR [30]. Alternative explanations for enrichment in coding exons could include changes in miRNA regulation or activation of degradation pathways, such as nonsense-mediated decay, which manifest as changes in steady-state mRNA level (see below) [31].

### Detection of *Trans-*eQTLs: Technical and Biological Complexities

Despite the prevalence of regulatory variation, most expression heritability is not explained by detectable eQTLs, suggesting that at many loci genetic variation in gene expression is driven by undetected variants of small effect [13], [18], [19], [32]. Some of this missing expression heritability may be explained by *trans*-acting regulatory variation [13], [18], [33], and indeed, *trans-*eQTLs do appear to have smaller effect sizes than their *cis* counterparts [20]. However, mapping *trans*-eQTLs has proved to be a challenging problem and many detected signals have replicated poorly [34]. The apparent small effect size of *trans*-eQTLs reduces power for detection [20], while the number of SNP–gene combinations required imposes a severe multiple testing penalty. These problems are compounded by small samples sizes in most eQTL studies. Computational methods to remove batch technical artefacts may also inadvertently remove *trans* eQTLs that affect large numbers of loci [35]. Regulation in *trans* may also be more tissue-specific and more difficult to detect in heterogeneous tissue samples [13].

In spite of these difficulties, recent large-scale studies have made progress in detection of *trans-*eQTLs [18], with some renewed support for spatial clustering of *trans*-eQTLs, for example near the MHC locus [36], [37]. However, even in these larger sample sizes, the numbers of *trans*-eQTLs detected has been modest and their replication rates have varied. Furthermore, although the search for *trans*-effects is frequently motivated by improved explanation of heritability of gene expression, the extent of this improvement remains unclear.

Although the small effect size of *trans*-eQTLs appears to be well supported, there may be a number of important exceptions to this rule. First, many *trans*-acting factors may function in signalling pathways and only activate in response to environmental stimuli. If so, large-effect *trans*-eQTLs may remain hidden when examining steady-state mRNA levels in quiescent cells, and there is some evidence that large effect *trans-*acting mutations only become apparent following cellular stimulation (B. Fairfax, personal communication) [39]. Second, it has recently been demonstrated that certain transcription factors, such as c-Myc, may act as “universal amplifiers” that increase the production of mRNA from thousands of genes simultaneously [40]. This result violates a standard experimental assumption that the total amount of RNA does not vary substantially from cell to cell across experimental treatments. One implication is that very large *trans-*effects could be missed when the same amount of RNA is extracted from all samples [41]. Although it seems unlikely that many such dramatic changes could be segregating as common variants in the population, it is possible that some large effect *trans*-eQTLs are missed altogether by this mechanism.

### Tissue-Specificity of eQTLs

eQTL studies in humans have been heavily biased toward LCLs. In some respects LCLs appear to be reasonable biological models. For example, eQTLs detected in LCLs are also found in primary tissues [42] and are highly enriched in disease associations [6], [7], which would be unlikely if most were artefacts of cell line transformation. However, gene expression levels in LCLs significantly correlate with other cellular traits, including EBV load and growth rate [43], and may exhibit monoallelic expression at some genes, which could reduce power to detect associations [44]. Perhaps most importantly LCLs are derived from a blood cell lineage (peripheral B-lymphocytes) and it is unclear how appropriate these cell lines are as models for nonblood or immune tissues.

Partly in response to this lack of cellular diversity, eQTL maps have been generated in a wider range of primary cells and tissues including whole blood, adipose tissues, primary B-cells, osteoblasts, monocytes, lymphocytes, skin, liver, and a variety of brain tissues [17], [21], [28], [45]–[49]. Many of these studies have focused on the cross-tissue replicability of eQTL signals. This is important because the level of replicability of eQTL signals between tissues determines whether an experimentally tractable cell line such as LCLs can be used as a proxy for other, less-accessible tissues. However, it is worth noting that “tissue-specificity” permits multiple interpretations including differences in effect size, allelic direction, or alternate effects on the same gene at unlinked SNPs [50], and these have not always been consistently defined across studies. Cross-tissue replication is also heavily dependent on the exact tissue comparison and on the extent of shared versus tissue-specific gene expression. Thus, although tissue-specificity of eQTLs is biologically plausible given the diversity of regulatory architecture across cell types, it is difficult to draw general conclusions from the results of individual studies.

One early report suggested that tissue-specificity of eQTLs was widespread, with only 20%–30% of signals observed in one tissue replicating successfully when tested in an alternative [28]. However, subsequent work has highlighted how correction for low power can increase overlap in eQTLs from different tissues substantially (from between 30% and 50% to approximately 70%) [51]. A recent reanalysis of the data set in [28] using a method that explicitly models eQTL-sharing across tissues has suggested that the true overlap of eQTLs across the three tissues in this data set is closer to 63% (http://arxiv.org/abs/1212.4786). More generally, results from multiple studies have estimated that replicability of eQTLs across tissues typically varies between 40% and 80% with more similar tissues unsurprisingly sharing a greater fraction of common eQTL signals [17], [18], [45], [50], [52], [53], although this relatively high degree of cross-tissue sharing has been disputed by some [13], [14].

### eQTLs in Disease and Complex Traits

In the majority of cases, human disease and complex trait association studies do not identify plausible coding variants implicating a single gene. As a result, post hoc identification of the causal gene(s) that underlie an association signal remains a significant problem that can limit the biological interpretability of disease association study results. However, eQTLs are by definition associated with a specific gene, and trait-associated variants that are also eQTLs can identify potentially causal genes for further functional studies. An early example of the power of this approach was highlighted by the discovery that asthma-associated variants spanning a region of 206 kb (and several gene loci) on the long arm of chromosome 17 [54]. A subsequent eQTL mapping experiment revealed that associated variants were also correlated with expression changes at a specific gene, ORMDL3. Further follow-up studies have suggested that changes in expression of ORMDL3 alter endoplasmic reticulum-mediated calcium signalling, which in turn may effect a change in inflammatory response [55]. Driven by the success of this and other studies, eQTL mapping is now becoming a standard tool for the identification of the genes and regulatory networks that are important for phenotypic variation, with recent applications to psoriasis susceptibility [51], psoriatic arthritis [56], LDL cholesterol levels [57], schizophrenia susceptibility [58], Type 2 diabetes [59], and obesity-related-traits [17] (for a comprehensive review, see [60]).

In summary, eQTL studies have demonstrated that the influence of common genetic variation on gene expression in humans is widespread. Genetic variation in gene expression likely results from the combined action of small numbers of relatively large effect *cis-*acting mutations, which are characteristically enriched near the regulated gene, and a more polygenic *trans* component that mostly eludes detection. The biological mechanisms that drive expression variation, however, remain less clear.

## Understanding Mechanism with Alternative Molecular Phenotypes

**Figure 1:**
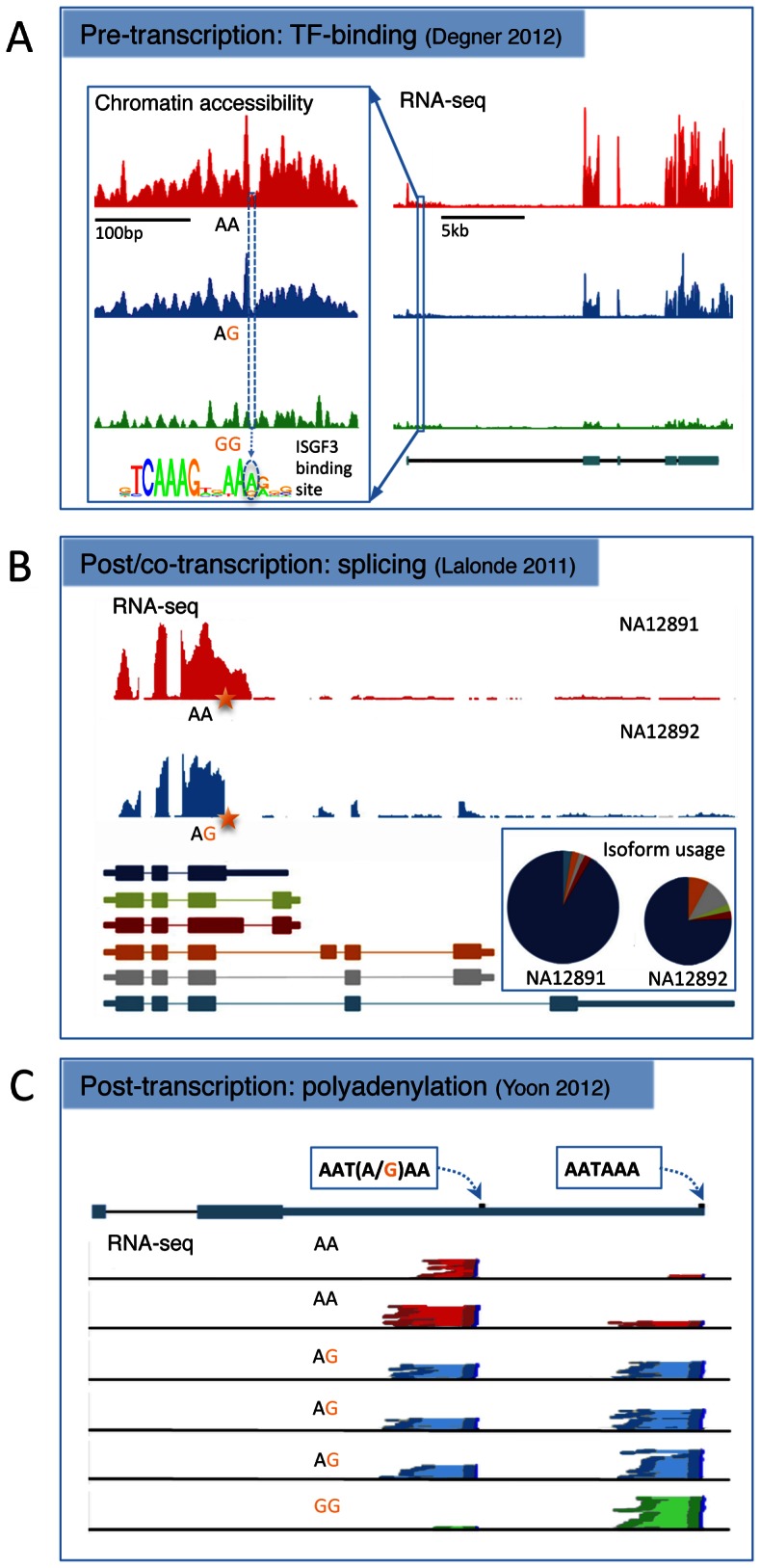
Three examples of alternative biological routes to gene expression variation identified using alternative molecular phenotypes from the recent literature. (A) A joint DNase-sensitivity/eQTL in the gene SLFN5 from [62]. The left-hand panel shows the landscape of open chromatin in a region ∼10 kb upstream of the gene TSS across 70 individuals grouped according to genotype at SNP rs11080327. The SNP is located in an interferon stimulated response element (inset), a TFBS that binds a range of related immune response TFs. The right-hand panel shows RNA-seq read depth across the transcript region, with the gene annotation from ENSEMBL underneath. This plot has been shortened slightly from the original for formatting reasons. Adapted with permission from [62]. (B) A splice variant that alters both the expression level and relative isoform abundance in the gene MRPL43 from [31]. The top panel shows RNA-seq read depth in two individuals that are homozygous or heterozygous for a SNP (rs2863095) immediately downstream of a splice donor site in exon 3. Below are shown the transcript annotations inferred from RNA-seq. The pie chart shows the relative isoform abundance usage in the two individuals, while the height of the pie chart reflects overall gene expression, summed over all transcripts. The star shows the location of the splice polymorphism in the transcript. Adapted with permission from [31]. (C) An example of a SNP (rs10954213) that alters 3′ polyadenylation site usage in IRF5 from [91]. Shown is the transcript annotation with the two alternative polyadenylation sites used, and the 3′ paired end RNA-seq data from six individuals with read pairs from each genotype colored dark red, blue, or green and the intervening sequence fragment colored pale red, blue, or green. Adapted with permission from [91].

Functional characterisation of eQTLs remains a significant challenge. Associated SNPs are usually spread over many kilobases, and because long-range regulation of gene expression is not uncommon, it can be difficult to prioritise individual variants based on their genomic location. In addition, steady-state mRNA levels are a function of a diverse set of molecular processes, any one of which can be affected by a given mutation (Figure 1). One approach to understanding this complexity is to extend mapping to additional molecular phenotypes, such as splicing, methylation, or transcription factor binding. Next-generation sequencing has made a variety of alternative molecular phenotypes accessible, and recent studies have begun to highlight the diversity of biological processes that regulatory variants may perturb.

### Pre-transcriptional Regulatory Variation

#### Transcription factor binding and chromatin structure

The rate at which pre-mRNA is produced can be regulated at any one of a series of steps prior to transcription (for a detailed review, see [61]), and there is some evidence that a large fraction of regulatory variation may be active at this stage of gene expression [31], [62]. Because many pretranscriptional processes are mediated by the binding of transcription factors to regulatory motifs, many such variants likely alter the binding activity of these factors, either directly or indirectly. Assays of transcription factor (TF) occupancy are therefore a key alternative molecular phenotype for understanding the biological basis of gene expression variation.

A common experimental approach for measuring occupancy genome-wide is chromatin immunoprecipitation followed by hybridisation with a microarray or high-throughput sequencing (ChIP-chip or ChIP-seq). Although powerful, ChIP-seq is not feasible for most transcription factors, as it requires an extremely high-quality antibody. An alternative is to assay molecular features, such as chromatin accessibility or histone modification, which are common to many regulatory regions regardless of exactly which transcription factors are bound [63], [64].

Recent work has established that changes in TF binding driven by genetic differences between individuals are relatively common and can be reliably detected using sequencing-based assays. Ref [65] used ChIP-seq for Ste12, a yeast transcriptional activator, in an experimental cross and detected genotype binding associations at ∼21% of variable binding regions. In humans, significant interindividual variation and allele-specificity has also been detected at between 7% and 11% of NFkB and CTCF binding sites in LCLs using ChIP-seq [66], [67]. A study of 24 TFs and a transcriptional co-activator in a single individual estimated that 5.5% of all binding sites containing a heterozygous SNP show significant allele-specificity [68]. Chromatin states are also clearly influenced by common genetic variation. Histone modifications show significant familial clustering and allele-specificity in human pedigrees [67], [69], and genetic associations with chromatin openness (DNase-sensitivity QTLs or ds-QTLs) have been detected at thousands of loci [62].

Association mapping of TF-binding offers significant improvements in variant localisation and biological interpretability over eQTL mapping. From the perspective of complex-trait studies, TF-binding or chromatin accessibility QTLs could enable the identification of causal mutations and point to the upstream processes that they disrupt to produce a disease phenotype. For example, it has been estimated that 56% of causal chromatin accessibility QTLs are found within the open chromatin itself, a region usually hundreds of base pairs in size [62]. Maps of open chromatin QTLs therefore offer a mapping resolution orders of magnitude higher than the mean size of a linkage disequilibrium block in humans.

In addition to improved resolution, association mapping of TF binding can enhance functional interpretation, in particular for those TFs with an associated position weight matrix (PWM). Evidence to date suggests that TF binding QTLs are strongly enriched in the canonical binding motifs of their cognate factors, suggesting many function by altering binding directly at the point of protein–DNA contact [65]–[67], [70]. Likewise, chromatin accessibility-associated variants are highly enriched in DNaseI footprints that also precisely mark the sites of protein–DNA interaction [62]. Changes within binding motifs also appear to alter TF occupancy or chromatin openness in the direction expected under the appropriate PWM model (Figure 1A) [62], . These results suggest that it will soon be possible to understand regulatory variation at the level of the DNA sequence.

Variants associated with changes in chromatin accessibility and TF occupancy also appear to explain downstream expression changes at nearby genes in an interpretable fashion. Allele-specific TF occupancy is substantially enriched near genes with eQTLs [68]. Similarly, ds-QTLs are 450-fold more likely to also be associated with changes in gene expression than a random SNP and a substantial fraction of eQTLs (55%) are also ds-QTLs (Figure 1A) [62]. The relationship between ds-QTLs and eQTLs also depends on genomic architecture in ways that illuminate underlying biological mechanisms. For example, the probability that a ds-QTL is also associated with gene expression change depends on, among other factors, whether open chromatin and gene TSS are separated by an insulator element and on the distance of an enhancer mark to the TSS [62].

Despite this progress, these studies have also highlighted our incomplete understanding of the regulatory code underlying transcription factor binding. For example, [68] demonstrate that many allele-specific differences in TF occupancy cannot be explained by variants in the canonical binding motif, suggesting a possible role for alterations in co-factors or chromatin structure. Clearly, future studies will need to incorporate these additional aspects to fully understand variation in TF-binding between individuals.

#### CpG methylation

Transcriptional silencing is frequently associated with CpG hypermethylation of gene promoters. In mammals, allele-specific CpG methylation is synonymous with genomic imprinting and X inactivation. However, genome-wide assays of CpG methylation have revealed an abundance of allele-specific methylation in humans extending beyond the modest number of known imprinted loci [71]–[74]. Similarly F1 crosses in mice have revealed hundreds or thousands of differentially methylated regions, many of which correlate with segregating genetic variation [75], [76]. More recently, association studies of genome-wide methylation in human brain tissue and LCLs have detected many significant associations between genotype and methylation level (methylation QTLs, or meQTLs), the majority of which appear to be in *cis* and close to the site of methylation [48], [77], [78]. Differential methylation appears to occur across multiple neighbouring CpG sites in many cases, suggesting correlated effects of genetic variation across a relatively large area [76]–[78]. Most studies have not assessed to what extent variable methylation is due to polymorphism at the CpG sites themselves versus at other sites, although this may contribute a substantial fraction of the variation in allele-specific methylation [74]. The immediate sequence context of the methylated cytosine may also play an important role in determining the impact of variant [76].

The biological mechanism whereby variable methylation alters gene expression is not entirely clear. CpG methylation may regulate gene expression directly, by blocking the access of transcription factors to the DNA or by binding of methyl-CpG-binding proteins that drive chromatin remodelling and compaction. Alternatively, changes in methylation levels may passively reflect other regulatory processes such as transcription factor eviction [79]. Some fraction of changes in DNaseI sensitivity do manifest at the level of methylation [79], and it has been estimated that approximately 30% of dsQTLs also manifest as meQTLs, suggesting some overlap in mechanism [62]. In this case, methylation assays may be extremely useful as proxies for other phenotypes because DNA extraction for methylation analysis often requires smaller amounts of material, and is less laborious, than methods such as ChIP-seq or DNaseI-seq. However, although meQTLs are strongly enriched for associations with mRNA levels, a relatively small fraction of eQTLs appear to be explained by changes in methylation [77], [80]. Many sites of allele-specific methylation appear to occur in isolation in the genome with minimal downstream impact on gene expression [76]. In addition, in the absence of additional molecular phenotypes, methylation variation may be less amenable to functional interpretation.

### Co/Post-transcriptional Regulatory Variation

#### Splicing

Splicing is the removal of transcribed introns from the pre-mRNA by a complex of small nucleolar RNAs and proteins known as the spliceosome, and is one of several important regulatory processes occurring during and after transcription. The development of splicing-oriented microarrays with probes targeting individual exons, and more recently, direct high-throughput sequencing of cDNA libraries, has enabled the study of transcript processing at a genome-wide level [81].

Detection of splicing-associated variants poses a number of unique challenges, and a consensus has yet to be reached on the optimal analysis strategy. A key issue is differentiating pre-transcriptional regulatory variation, which alters transcription rate uniformly over *all* isoforms of a gene, from post-transcriptional variants, which alter the *relative* abundances of a set of isoforms and could be more correctly described as splice QTLs (sQTLs). Current technologies cannot measure individual isoform abundances directly and so differences in isoform expression levels must be inferred from individual exons, while also accounting for uniform changes across all expressed transcripts. This is further complicated by the fact that alternative isoforms may not share the same TSS. Approaches to this problem have varied widely and comparisons across studies are therefore problematic. A second issue is that, so far, association studies of splicing variation have featured relatively small sample sizes and have performed a substantially larger number of tests than eQTL mapping experiments. As a result, multiple testing thresholds are often severe and the numbers of sQTLs reaching genome-wide significance are modest.

Early applications of exon array technology suggested that alternative splicing of an exon between individuals is heritable and relatively commonplace (occurring in 1%–5% of measured exons) [82]. Mapping of sQTLs has now been performed in a variety of experimental settings, with significant associations (controlling study-wide FDR at between 5% and 10%) detected in roughly 10% of genes assayed [26], [45], [83]–[86].

Compared with whole gene expression, splicing variation has proved slightly more amenable to functional interpretation. sQTLs are enriched close to, or within, the spliced exon itself [26], [85], within the intronic binding sites of splice factors and in the canonical donor and acceptor splice sites [26]. Alternatively spliced exons also appear to be nonrandomly distributed across the transcript, with some evidence of enrichment toward the 3′ UTR [83], [85], [86], although this may reflect biases in sequence coverage due to the larger size of the 3′ UTR relative to internal exons. The most common form of splice variation appears to be simple exon skipping, with more exotic varieties such as mutual exon exclusion and intron retention detected in a small fraction of cases [86]. In addition to changes in the ultimate protein product, transcript compositional change can also indirectly influence total mRNA levels. Ref. [31] presents a series of examples where genetic variation in splice sites effects a post-transcriptional change on both relative isoform abundances and total mRNA levels via altered splice site efficiency, activation of nonsense-mediated decay, or SNPs in the 5′ UTR altering RNA stability (Figure 1B).

#### miRNAs, polyadenylation, and mRNA decay

Following RNA processing, the fully mature mRNA molecule is subject to spontaneous and directed degradation, possibly via interaction with small RNAs. Genetic variation affecting either mRNA stability or small RNA activity can alter the rate of mRNA decay and, ultimately, change steady-state mRNA levels. Although this area is still poorly understood, recent work has begun to examine the importance of these pathways in explaining interindividual expression variation, again focussing on human LCLs as a model system [87]. After treatment of cells with an RNA elongation inhibitor, they used a time-course of mRNA extractions to examine the decay rate of all expressed protein coding genes and detected joint RNA-decay (rdQTLs) and gene expression QTLs at 195 loci. They estimated that up to 19% of eQTLs could be explained by variation in mRNA decay rates and the within-gene distribution of associations suggests that many joint QTLs function by altering miRNA binding sites. Surprisingly, however, almost half of the detected associations in this study unexpectedly showed a positive correlation between decay rates and expression levels, in opposition to the intuitive model where higher decay rates lead to lower expression.

This result hints at an unappreciated degree of complexity underlying post-transcriptional regulation of steady-state mRNA levels. Despite the results of [87] and although miRNAs are well-established as negative regulators of gene expression, their role in mediating interindividual protein-coding expression variation remains unclear. A number of studies have also investigated regulatory variation directly affecting miRNA expression levels using either miRNA-specific microarrays or RNA-seq adapted for small RNAs [88]–[90]. The fraction of miRNAs showing a significant association between expression level and genotype appears to be approximately the same as for protein-coding genes in studies of equivalent size [89]. However, the statistical thresholds used to identify some miRNA eQTLs have been relatively liberal, and given the relatively small number of miRNAs that are expressed in a given tissue (<500), the absolute numbers of significant associations detected are small (<20). In addition, it is unclear how important interindividual variation in miRNA levels is for explaining population variation in protein-coding gene expression. A study of adipose tissue from 131 individuals did not find a negative correlation between the expression levels of miRNAs and protein-coding gene targets [89]. This study also suggested substantial experimental artefacts in the RNA-seq protocol used, suggesting further technical development may be required before the true importance of miRNA eQTLs can be established.

An additional path by which polymorphisms can affect mRNA stability is via alternative polyadenylation. Following splicing, mRNA is cleaved and multiple adenines are added to the 3′ end. Polyadenylation is important in preventing the mature mRNA from undergoing degradation, and polymorphisms interfering with this process could affect the efficiency of transcription and ultimately lead to variation in mRNA half-life. In addition, alternative polyadenylation can provide different substrates within which further regulation, for example by miRNAs, can occur. Ref. [91] examined variation in polyadenylation in six LCLs using RNA-seq and found that mutations located in the 6 bp polyadenylation signal sequence were significantly more likely to be associated with gene expression variation relative to mutations occurring in the 3′ UTR overall. In a series of experiments, this study demonstrated that changes in adenylation appeared to confer alternative stability on different transcript isoforms, possibly via the differential inclusion of certain regulatory motifs, such as AU-rich elements or miRNA binding sites, in the mature transcript. An example of a regulatory variant affecting polyadenylation is given in Figure 1C.

In summary, studies of alternative molecular phenotypes are beginning to reveal the biological mechanisms of gene expression variation. Results to date suggest that a relatively large fraction of gene expression variation results from genetically driven changes in transcription factor binding, indicating a regulation at the pre-transcriptional stage. Some fraction of these changes is also reflected epigenetically at the level of CpG methylation, although the extent of this is unclear. Changes in splicing are relatively common and may be more tractable phenotypically, while the contribution of other post-transcriptional regulation is currently uncertain.

## Conclusions and Future Directions

Genetic mapping of molecular and cellular traits has an important future role to play in human genetics and genomics. In this section I will highlight some areas for future development.

### Incorporating Function and Progress toward Predictive Models

Despite progress using alternative phenotypes, the majority of eQTLs have resisted interpretation at the level of DNA sequence. A key goal for future studies will be the incorporation of more explicit models of gene regulation into the mapping of regulatory variants. Some progress has been made in this direction by the development of prior models that incorporate functional data [27], [29], [92], but there is much scope for improvement. Incorporation of regulatory models in eQTL mapping is attractive for two reasons. First, explicit regulatory models naturally allow for the inclusion of additional functional data in association mapping. This can substantially improve prioritization of individual SNPs for functional follow-up and is particularly useful for interpretation of disease associations and rare noncoding variation. Second, this approach provides a natural framework within which to develop and test models of gene regulation using naturally occurring genetic variation as an experimental perturbation. This is a powerful approach for understanding the regulatory code and, ultimately, for the development of models to predict the location and impact of regulatory variants.

### Expanding Alternative Phenotypes

High-throughput sequencing continues to be appended to standard molecular biology techniques, and many different aspects of gene regulation can now be quantitated using read-coverage metric. These developments offer the opportunity to genetically map highly specific molecular processes and dissect gene regulation on ever-finer scales. For example, recent developments such as GRO-seq [46] or ChIP-exo [47] could potentially be extended to extremely high-resolution QTL maps of productive mRNA elongation or TF-binding. Likewise, as others have highlighted, association mapping will continue to be extended to high-level cellular phenotypes, improving our understanding of the downstream consequences of changes in gene expression [4]. Quantitative, high-throughput measurement of protein expression levels will be of particular importance for understanding the functional significance of changes at the mRNA level. Although progress has been slow, recent developments suggest that detection of protein QTLs may soon be possible [93]. Finally, as more exotic species of noncoding RNA continue to be discovered, the application of eQTL mapping to, for example, long noncoding RNAs is likely to reveal deeper insights into the importance of these molecules for phenotypic variation and disease.

### Novel Cellular Environments and Inaccessible Tissues

A significant limitation of most cellular association studies to date has been the restriction to cells in a steady or quiescent state. In fact, many phenotypes of interest, in particular those that represent a response to environmental or pathogen stimulus, are likely to remain hidden in this system. An important area for future work will be detection of variant–environment interactions, with a particular focus on stimulation of cells using pathogenic or other stress factors. Work in this area has already begun to examine the effects of a variety of environmental stresses, including radiation and exposure to drugs, steroids, or pathogens, on maps of regulatory variation [39], [43], [94], [95]. Related to this, despite many efforts to expand the cellular repertoire of eQTL maps beyond LCLs and large-scale initiatives such as the Genotype-Tissue Expression program (http://commonfund.nih.gov/GTEx/), some cell types are always likely to remain inaccessible by conventional methods. The advent of induced pluripotent stem cells will enable the collection of stem cell lineages from large numbers of healthy individuals and allow for extension of genetic maps of regulatory variation into early development and difficult-to-sample cell lineages, such as live neurons.

### Standardised Analysis and Data Release

The utility of cellular QTL mapping studies, both for human genetics and genomics, strongly depends on the availability and quality of the data produced. However, despite some efforts at creating a centralised database (e.g., [96] or http://eqtl.uchicago.edu/cgi-bin/gbrowse/eqtl/), the results of many studies are not easily accessible. This inaccessibility is driven primarily by the lack of a standardised analysis framework and the failure of many studies to release their full data (genotypes and molecular traits) into the public domain. Development of widely appreciated analysis methods, quality control, and replication criteria has been enormously beneficial for human disease mapping. In contrast, as others have also highlighted [23], comparison and interpretation of results from multiple eQTL mapping studies, even those performed in the same tissue, is complicated by wide variation in statistical thresholds and analysis pipelines implemented. Compounding this problem is the lack of availability of raw data for reanalysis and methods development. The 10 years since the first human eQTL mapping studies have seen dramatic improvements in the statistical analysis of gene expression data (e.g., [97]). This offers an attractive opportunity for methods development and reanalysis of previous studies, in particular those performed in novel or difficult-to-obtain tissue types. It is unfortunate that, while most groups release raw gene expression data, many do not release genotype data because of concerns regarding confidentiality. While most studies do publish lists of significantly associated SNPs, this is far from optimal given the wide variation in analysis methods. A possible solution would be the use of resources such as the European Genotype Archive (https://www.ebi.ac.uk/ega/) that allow for managed access to published data sets, but still facilitate sharing of raw data. Failing this, emphasis on standardised analysis would at least facilitate fair comparison and collation across studies. Greater emphasis on public access of the datasets created for cellular association studies can greatly improve the impact and uptake of results produced. Ultimately association studies with cellular traits should enable greater functional interpretation of the mechanisms of phenotypic variation and disease, and efforts to facilitate this should be strongly encouraged.
